# Current surgical techniques for the management of pediatric glaucoma: A literature review

**DOI:** 10.3389/fopht.2023.1101281

**Published:** 2023-03-22

**Authors:** Zeynep Aktas, Gokcen Deniz Gulpinar Ikiz

**Affiliations:** ^1^Department of Ophthalmology, Atilim University School of Medicine, Ankara, Türkiye; ^2^Department of Ophthalmology, Medical Park, Ankara, Türkiye

**Keywords:** pediatric glaucoma, trabeculectomy, trabeculotomy, minimal invasive glaucoma surgery, glaucoma surgery

## Abstract

Pediatric glaucoma surgery is challenging due to its diverse and complex pathophysiology, altered anterior segment anatomy, greater potential for failure, and complications compared to adult patients. Moreover, numerous challenges are associated with long-term postoperative management. Thus, when dealing with childhood glaucoma, it is important to consider the potential complications in addition to the benefits of each intervention. The purpose of this article is to review recently published literature to shed light on the most recent surgical techniques for the safe and effective treatment of childhood glaucoma. Current literature shows that goniotomy and trabeculotomy are the first choices for the management of primary congenital glaucoma. Although older children with phakic eyes seem to benefit from trabeculectomy with adjunctive mitomycin C, it carries a long-term risk of bleb-related endophthalmitis. Glaucoma drainage devices may be preferred for patients with secondary or refractory glaucoma. However, hypotony or tube-related complications are common and encountered more often in children than in adults. Cyclodestructive procedures are also an option for cases in which filtering surgery has failed, but they can also be used as a temporizing measure to reduce the rate of complications in high-risk patients. However, their outcomes can be unpredictable, in terms of efficiency and complications. Finally, minimally invasive glaucoma surgery (MIGS) as the sole alternative treatment or as an adjunctive surgical procedure is a relatively new path for pediatric patients.

## 1 Introduction

Glaucoma is a specific type of progressive optic neuropathy caused by increased intraocular pressure (IOP), which is associated with characteristic visual field changes leading to irreversible vision loss. According to the Childhood Glaucoma Research Network (CGRN) classification system, primary childhood glaucoma includes juvenile open-angle glaucoma and primary congenital glaucoma (PCG), while secondary childhood glaucoma includes glaucoma following cataract surgery, glaucoma associated with non-acquired systemic disease or syndrome, glaucoma associated with non-acquired ocular anomalies, and glaucoma associated with acquired conditions (1, 2).

Childhood glaucoma leads to irreversible vision loss due to corneal edema, Haab striae, amblyopia, and progressive optic nerve damage. The best way to avoid these consequences is to control IOP. Multiple examinations under anesthesia are generally required to check axial length, corneal diameters, corneal clarity, and optic nerve appearance in addition to IOP (3). Medical treatment is an important component of the management, depending on the type of glaucoma. However, surgical treatment is critical in most of the cases because of associated anatomical abnormalities in the trabecular meshwork and Schlemm’s canal (1, 4, 5). Goniotomy and trabeculotomy, either in a traditional or circumferential manner, constitute angle surgery and are the preferred initial treatments for patients with PCG. Trabeculectomy can also be performed together with trabeculotomy or when angle surgery fails. Glaucoma drainage devices (GDD) are generally preferred in patients with refractory childhood glaucoma and often provide effective long-term IOP control (1). Cyclophotocoagulation might not be as effective in the long-term and it frequently necessitates additional sessions without cessation of medical therapy (1). Micro-invasive glaucoma surgery (MIGS), consisting of novel surgical procedures using new glaucoma microstents, has recently been proposed as a primary procedure or adjunctive to other procedures for refractory primary or secondary childhood glaucoma. When dealing with childhood glaucoma, it is important to consider the potential complications in addition to the benefits of each intervention. The purpose of this article is to review the recently published literature focusing on the most recent surgical techniques for the safe and effective treatment of childhood glaucoma.

## 2 Angle surgery (goniotomy/trabeculotomy)

### 2.1 Goniotomy

Otto Barkan described a technique that makes a 90-120° nasal incision (6). For the safety of this procedure, visualization of intraocular and angular structures is essential (1, 6). This surgery might not be suitable for patients with cloudy corneas. In cases with diffuse epithelial edema, peeling off the epithelium can be sufficient; however, stromal edema will not resolve with this intervention and might still be a problem.

**Figure 1:**
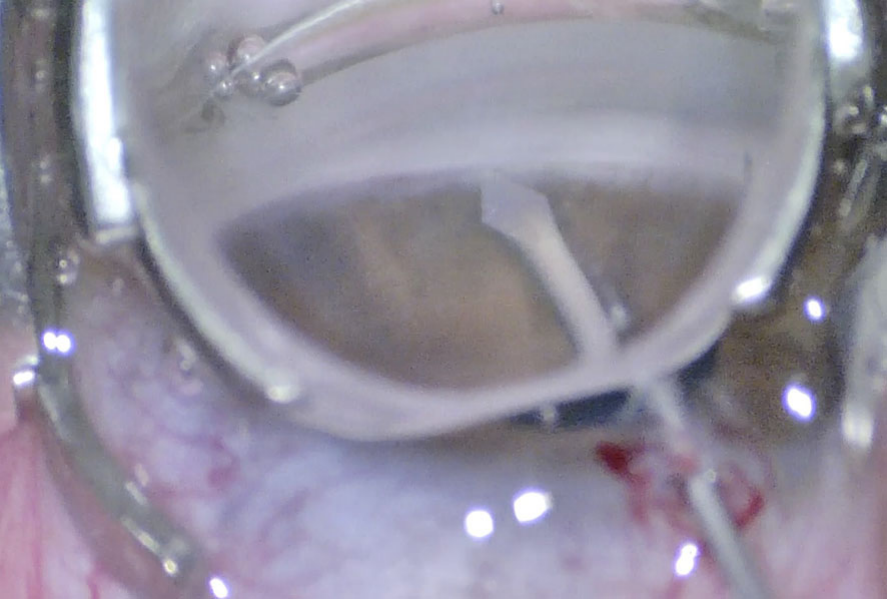
Goniotomy performed with a microvitreoretinal (MVR) blade in a patient with primary congenital glaucoma.

Preoperative medications are recommended to substantially reduce the IOP. This is essential for enhancing corneal clarity and providing an optimal angle visualization. Direct goniolenses are utilized for effective visualization of the angle details (Swan-Jacobs goniolens, Khaw goniotomy lens, or Barkan lens). A short tunnel is created with a blade or needle (23G/25G needle) through the temporal cornea to avoid iris prolapse (1). The anterior chamber (AC) is maintained using a viscoelastic material or AC maintainer. Subsequently, the instrument is directed upward when it enters the AC to prevent damage to either the iris or lens. Furthermore, preoperative topical use of pilocarpine (1-2%) might be considered to provide pupil constriction for the protection of the lens against trauma. The blade is hold at an angle halfway between the iris root and the Schwalbe line and is moved circumferentially along the angle, creating an angle incision nasally. Subsequently, a cleft with the exposure of less pigmented tissue is seen, accompanied by enlargement of the angle and posterior iris movement (**Figure 1**). Following withdrawal of the knife or needle, blood reflux often occurs and usually resolves when the AC is formed and IOP is elevated with a balanced salt solution. Bleeding might be further reduced by topical administration of 0.5% apraclonidine or diluted intracameral epinephrine (1:16,000) (1, 6). Goniotomy *via* incision of the trabecular meshwork results in less resistant access to Schlemm’s canal and increases aqueous outflow.

The first long-term reports on goniotomy revealed 80–90% overall success rates in children with PCG presenting between 1 month and 1 year of age (77–9). In children presenting at birth or within the first month neonatally, success rates decreased down to 30–50% (5, 7). Hypema is reported to be one of the most common complications, which generally subsides without any intervention within the first postoperative week. Peripheral anterior synechiae, iridocyclodialysis, injury to the crystalline lens, and retinal detachment in highly myopic eyes are other relatively rare complications (8).

The success rate after goniotomy varies from 30% to 90% in various reports, depending on the timing of diagnosis, the severity of the disease at presentation (IOP on baseline, axial length at presence), the presence of corneal cloudiness at presentation, and the number of goniotomies required (7, 9–11). Shaffer noted that PCG had the best outcomes where there was an isolated trabeculodysgenesis. Many cases presented at birth could have been diagnosed as iridotrabecular dysgenesis, as many have hypoplasia and increased iris vascularization, which is related to poor prognosis. Moreover, patients over 2 years of age had more collagenous tissue, which may be responsible for the low success rates in this group (7). The overall success rate of goniotomy in PCG reported in recent studies, regardless of the time of diagnosis, also fits in this range, confirming that late-onset PCG has a poorer prognosis than non-PCG cases (**Table 1**). Agarwal et al. reported that 80% of congenital glaucoma cases in India presented with hazy corneas, due to the delay in diagnosis, which was associated with poor visual prognosis (15). Mandal et al. reported promising outcomes with combined trabeculectomy-trabeculotomy (CTT), which is one of the most preferred surgical techniques in India for the treatment of childhood glaucoma (16, 17). In contrast, Kaushik et al. reported a higher success rate in India than those reported in Western studies (**Table 1**) (14).

**Table 1:** Studies on goniotomy for primary congenital glaucoma.

Year	Author	Surgical technique	Eyes	Follow-up (years)	End point	Complete Success rate	Complication
1953	Barkan et al. (11)	Goniotomy	196	17	IOP<20mmHg	80.0%	N/A
1982	Shaffer (7)	Gonitomy	287	15	IOP<20mmHg	76.7%	Hyphema (0.3%)
							Iridodialysis (0.7%)
							Cyclodialysis (0.3%)
							Shallow anterior chamber (0.9%)
2018	Hassanein et al. (12)	Goniotomy	81	5.9	IOP<18mmHg	50.6%	N/A
2019	El Sayed et al. (13)	Gonitomy	120	0.5	IOP<18mmHg	41.0%	Shallow anterior chamber (4%)
							Choroidal detachment (0.8%)
							Iatrogenic cataract (2%)
2021	Kaushik et al. (14)	Goniotomy	31	2	IOP<19	67.7%	N/A

DMT, Descement membrane tear. N/A, Not available.

Studies also revealed that follow-up time is another factor affecting success rate, which is worth mentioning. In each individual study, the success rate decreased with time, which is an important problem to consider for lifelong follow-up of children with glaucoma.

Goniotomy failure in childhood glaucoma is often related to certain risk factors, including early intervention (as early as the first neonatal month) and positive consanguinity. Retreatment with goniotomy provides variable success (12). However, its value as an initial approach for complex glaucoma has not yet been clearly proven. Goniotomy can be performed in children with uveitic glaucoma after the subsidence of active inflammation. Bohnsack et al. reported the surgical outcomes in childhood uveitic glaucoma. Goniotomy was the initial surgery in 31 eyes, among which, 15 eyes achieved adequate control (IOP 18 mmHg with or without medication) 48 months following surgery independent of the lens status (phakic/pseudophakic/aphakia) (18).

Trabeculotomy should be considered in cases where visualization of the angle structures is inadequate. Peroperative endoscopic visualization of the angle in cases of hazy corneas has been described by certain reports in the literature (19).

### 2.2 Trabeculotomy

**Figure 2:**
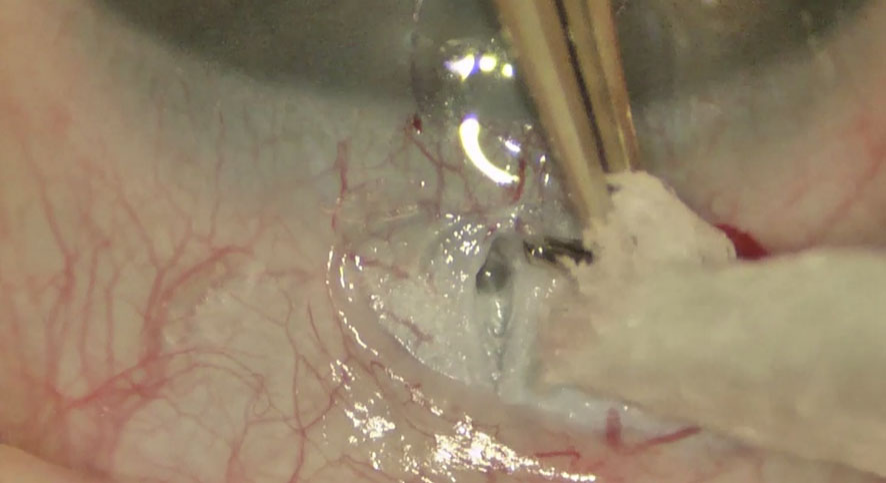
Aqueous reflux from the cut ends of the Schlemm canal.

**Figure 3:**
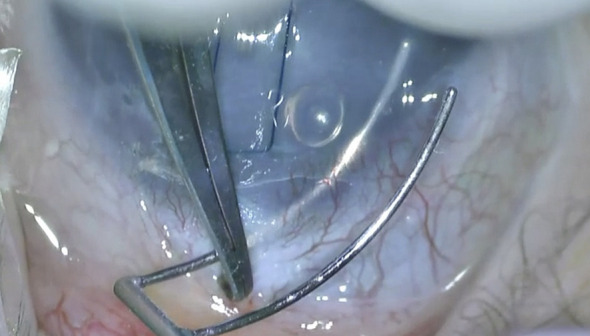
Introduction of the Harms trabeculotome probe into the Schlemm’s canal and and its rotation toward the anterior chamber.

In 1960, Redmond Smith described an external approach to the AC angle: a ‘nylon filament trabeculotomy’ in which trabecular meshwork is ruptured with a nylon suture. The conventional approach includes a metal trabeculotome probe, as described by Hermann Burian, and provides an incision of up to one-third of the angle (20, 21). A side port is initially created, which is used for maintenance of the AC during surgery. Following the creation of a partial-thickness scleral flap, Schlemm’s canal is detected under high magnification. The incision is extended deeper, more slowly, and carefully at the limbus. The inner wall of Schlemm’s canal is avoided to prevent inadvertent entry into the AC. An accurate incision of the Schlemm’s canal results in the reflux of blood or aqueous (**Figure 2**). The patency of the ostium is confirmed by probing with 6/0 nylon or polypropylene sutures (1). Then the trabectome probe is introduced and slightly rotated anteriorly toward to AC (**Figure 3**).

**Figure 4:**
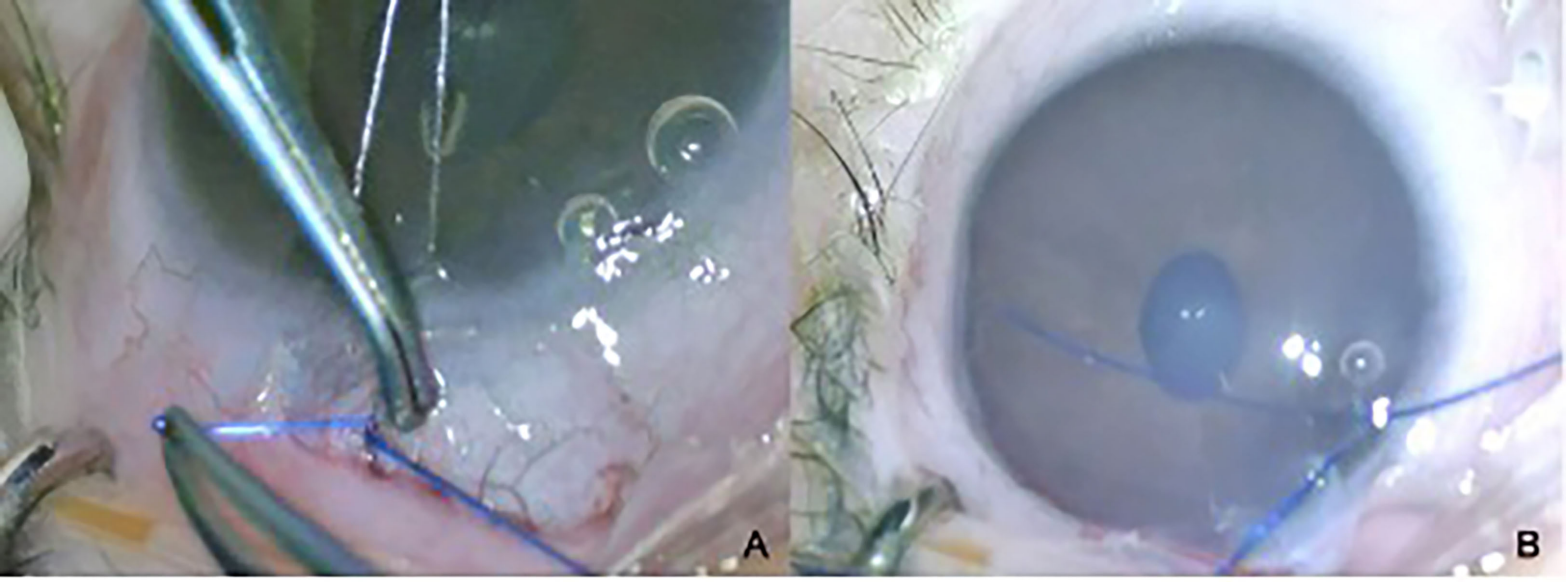
Completion of circumferenital advancement of 6-0 Prolene suture **(A)** and externalization of the suture *via* the side port to be pulled as an alternative method **(B)**.

There have been numerous recent variations of this traditional technique. Two-site rigid trabeculotomies result in the extension of the incision to 180—360°. Circumferential trabeculotomy is an alternative technique that enables a 360° incision of the angle using a blunted suture (i.e., 6/0 prolene) or an illuminated microcatheter, as described by Beck and Lynch in 1995 (1). Either the suture or the catheter is introduced into one of the incised ends of the canal and advanced through 360° until it exits *via* the ostium (**Figures 4A, B**). Subsequently, the instrument was carefully pulled to disrupt the trabecular meshwork. Another alternative technique is viscotrabeculotomy, in which the Schlemm’s canal is entered with a Grieshaber cannula and dilated on either side with adequate Healon GV injection prior to the introduction of the trabeculotome (22).

Traditional trabeculotomy (TT) in children following multiple surgeries has been shown to be at least as successful as goniotomy (13, 23–26). In studies comparing goniotomy with TT, the success rate seemed to be influenced by the grade and extent of the disease rather than the technique (27, 28). Ozawa et al. reported their study into TT, which included Japanese children under three years of age, two-thirds of whom were diagnosed with PCG (29). In eyes with failed initial trabeculotomy, repetitive procedures resulted in a success rate of over 90% at 5 years and up to 70% at 25 years (**Table 2**). The procedure did not achieve comparable success in children with secondary glaucoma when compared to PCG. TT has proven to have satisfactory success in patients with aphakic and uveitic glaucoma (33, 34). Wang et al. reported 81% success at 31 months after rigid trabeculotomy in eyes with uveitic glaucoma (33). Trabeculotomy can be performed repeatedly and has the advantage of low rates of postoperative hypotony (34).

**Table 2:** Summary various studies on surgical treatment for Primary Congenital Glaucoma (PCG).

Year	Author	Surgical technique	Eyes	Follow-up (years)	End point	Complete Success rate	Complication
2017	Ozawa et al. (29)	Trabeculotomy	68	25	IOP<22 mmHg	92%	N/A
2019	El Sayed et al. (13)	Trabeculotomy	332	0.7	IOP<18mmHg	58%	Shallow anterior chamber (4%)
							Iatrogenic cataract (3%)
							Lens subluxation (1%)
							Endophtalmitis (0.3%)
2020	Esfandiari et al. (28)	Trabeculotomy	63	7	IOP<22mmHg	65%	N/A
2016-2020	Dragosloveanu et al. (30)	Trabeculotomy	38	5	N/A	89.5%	Hyphema (78.9%)
2016-2020	Dragosloveanu et al. (30)	Trabeculotomy (360 degrees)	41	5	N/A	97.5%	Hyphema (82.93%)
2015	Shi et al. (31)	Trabeculotomy	15	1	IOP<21mmHg	51.6%	Hyphema (100%)
2015	Shi et al. (31)	Trabeculotomy (360 degrees)	16	1	IOP<21mmHg	81.0%	Hyphema (100%)
2020	Aktas et al. (32)	Trabeculotomy	25	2.9	IOP<22 mmHg	24%	Microhyphema (100%)
							Macrohyphema (20%)
							DMT (4%)
							Suprachoroidal haemorrhage (4%)
							Serous retinal detachment (4%)
2020	Aktas et al. (32)	Trabeculotomy (360 degrees)	17	2.9	IOP<22 mmHg	47.1%	Microhyphema (100%)
							Macrohyphema (5.8%)
							DMT (5.8%)

N/A, Not available.

Circumferential trabeculotomy (CT) has a 90% success rate, sustained for 4 years postoperatively, which is superior to TT (35–37). The success rate of two-site rigid trabeculotomy is also reported to be superior to that of TT, providing a 120° incision, which is comparable to that of CT. In a study comparing TT with CT in children with secondary glaucoma, Lim et al. reported that both procedures remarkably lowered IOP (**Table 3**) (38). However, CT resulted in significantly lower IOP and a higher success rate than TT (30). The predominant diagnosis in the study group was PCG despite the presence of pseudophakic glaucoma and glaucoma secondary to aniridia. The continuation of medical therapy was similar between the groups in the first postoperative year. The CT group required fewer additional glaucoma surgeries within the first year than the TT group (14% vs. 42%, respectively). When only cases with a diagnosis of PCG were compared, the success rate with TT was 55.7%, whereas it was 100% for CT. Aktas et al. reported the outcomes of TT and CT for the treatment of neonatal-onset PCG, which showed that the success rate of CT was superior (32). Combined nasal goniotomy and temporal trabeculotomy providing almost circumferential intervention of the angle is an alternative surgical technique for PCG, which was recently presented by Abdelrahman et al. (40) The early postoperative success rate was 93.3%, maintaining 60% success rate at 18 months. This technique seems to be a safe and effective surgical alternative for PCG, leaving the superior conjunctiva untouched for possible future interventions (40). Shi et al. compared microcatheter-assited CT with rigid probe and reported complete success rates of 81.0% and 51.6%, respectively (31).

**Table 3:** Studies investigating angle surgery in pediatric glaucoma following cataract surgery.

Year	Author	Surgical Technique	Eyes	Lens status (n)	Mean Age at Surgery	Follow-up (year)	End Point	Qualified Success Rate	Complication
2017	Lim et al. (38)	Trabeculotomy (microcatheter 360 degrees)	29	Aphakic (21)	5.6	2.6	IOP<23mmHg	72%	Vitreus Hemorrhage
2019	El Sayed et al. (13)	Trabeculotomy	15	Aphakic (16)	5.7	1.4	IOP<23mmHg	89.6%	Vitreus hemorrhage
		(Two site rigid probe 180-360 degrees)		Pseudophakic (13)					Progressive myopic shift
2020	Rojas et al. (39)	Trabeculotomy (microcatheter 360 degrees)	15	Aphakic (12) Pseudophakic (3)	7.8	3.3	IOP<21mmHg	93.3%	Vitreus hemorrhage
2019	El Sayed et al. (13)	Trabeculotomy	33	N/A	5.7	1	IOP<23 mmHg	89.6%	Vitreus hemorrhage
		(Two site rigid probe 180-360 degrees)							

## 3 Trabeculectomy

Trabeculectomy can be performed in children with secondary glaucoma or in those who previously had unsuccessful angle surgery (1). Conjunctival peritomy is created in a superior fornix-based manner, followed by the creation of a thick (5 × 4 mm) lamellar scleral flap with a crescent blade. Dissection is moved forward into the cornea to avoid the iris, ciliary body, and vitreous incarceration. Short radial cuts are made because of the elasticity of the child sclera, which provides closure without necessitating suturation of the radial edge of the flap and also allows posterior aqueous flow. Mitomycin C (MMC) is applied to a wide area of subconjunctival space and the undersurface of the scleral flap at concentrations varying between 0.1 and 0.5 mg/ml for 2-3 minutes before irrigation with a balanced salt solution. At the corners, 10-0 nylon scleral sutures are placed in a fixed manner, and two releasable sutures are placed at the posterior edge of the scleral flap. Paracenthesis is constructed with a 21 G needle, as an oblique, peripheral long tunnel to avoid disruption of the lens, provide a tight wound, and stabilize the infusion cannula. Sclerectomy is performed with a small sclerostomy punch (500-mm) or knife to allow better control of the aqueous outflow. It is also important to perform sclerostomy as anteriorly as possible to prevent incarceration of the iris, ciliary body, or vitreous. Subsequently, an iridectomy is surgically performed. Additional sutures are placed in the scleral flap at the end of the procedure. After closure of the conjunctiva with 10-0 nylon sutures, a small volume of viscoelastic and sterile air is often left in the AC.

Trabeculectomy provides satisfactory and sustainable success (65%-80%) and still counted as the gold-standard approach for eyes that have undergone unsuccessful angle surgery (31, 41). It can be performed either alone or in combination with trabeculotomy in children with a history of goniotomy failure (31, 41, 42). Jayaram et al. evaluated the long-term success and safety of trabeculectomy with MMC in children with PCG, aged less than 2 years, most of whom underwent failed goniotomy surgery (**Table 4**) (31). Jalil et al. reported their study on children treated with combined trabeculotomy-trabeculectomy (42). It revealed that, combined trabeculotomy-trabeculectomy (with 5FU) appeared to be an efficient procedure with complete success achieved in 19 out of 29 eyes (65.5%) at the end of 4 years. However, in cases of uveitic glaucoma secondary to juvenile idiopathic arthritis, nearly half of the cases failed by the end of the first year according to the outcomes of the another study by Wiese et al. (43) Thus, trabeculectomy is also more effective in PCG than that of secondary childhood glaucoma (31). Trabeculectomy may be preferred over GDD in cases of anterior segment dysgenesis, as it provides efficient IOP control and has the advantage of being potentially less detrimental to the corneal endothelium (1, 44).

**Table 4:** Summary of trabeculectomy in childhood glaucoma.

Year	Author	Surgical technique	Eyes	Follow-up (year)	End Point	Complete Success Rate	Complication
2011	Jalil et al. (43)	Trabeculotomy+Trabeculectomy with 5FU	29	3.4	IOP<22mmHg	65.5%	Blebitis (3.4%)
							Hyphema (24%)
							Choroidal detachment (3.4%)
2015	Jayaram et al. (41)	Trabeculectomy with MMC	40	5.2	IOP<22mmHg	62.5%	Choroidal effusion (10%)
							Blebitis (2.5%)
2018	Hsu et al. (42)	Trabeculotomy+Trabeculectomy with MMC	65	15	IOP<21mmHg	81.3%	Hyphema (12%)

MMC, Mitomycin C.

5FU, Fluorouracil.

Potential complications of trabeculectomy are encountered in the short-term postoperative period and are usually associated with hypotony (shallow AC, choroidal effusion, hypotony maculopathy, and suprachoroidal hemorrhage). Thinning of the bleb wall and formation of cystic blebs (inflammation of the bleb, endophthalmitis, and leakage of the bleb) are potential complications that could be encountered later. Hyphema is an innocuous perioperative complication that often resolves spontaneously.

## 4 Deep sclerectomy

Deep sclerectomy is a filtration procedure in which the internal wall of Schlemm’s canal is removed, leading to exposure of the trabecular meshwork. Although it is proposed as an alternative to penetrating filtration procedures, it can be applied in combination with trabeculotomy/trabeculectomy (45). Tixier et al. showed a success rate of 75% after deep sclerectomy in 12 eyes of eight patients with congenital glaucoma (46). However, Lüke et al. reported that complete success was not achieved in any case when undergoing deep sclerectomy alone from among a relatively small group (four of 10 procedures). (47) Lack of a clear identification of Schlemm’s canal in four eyes (40%), choroidal deroofing in one eye (10%), and visible perforation of the trabeculo-descemetic membrane in two eyes (20%) were the complications reported in this study. The success rates of combined procedures in literature are more consistent. Feusier et al. reported a complete success rate of 52.3% with the “penetrating deep sclerectomy” (combined deep sclerectomy and trabeculectomy) (48). Bayoumi compared the efficacy of combined trabeculotomy—trabeculectomy with mitomycin C (CTTM) and CTTM with deep sclerectomy for the treatment of PCG (45). The study covered 20 eyes of 20 patients and revealed that the success rates were similarly high, and the addition of deep sclerectomy to the procedure of CTTM in pediatric glaucoma surgery shortens the duration of surgery and is not associated with additional complications (45).

## 5 Glaucoma drainage devices

GDD is an important alternative in childhood glaucoma, especially in refractory cases in which the progression of the disease cannot be controlled by previous angle surgery/trabeculectomy or as an initial surgical preference in secondary childhood glaucoma.

Molteno was the first to describe the use of GDD in pediatric patients in 1973 (49). The design of all GDDs aimed to create a shunt for the aqueous solution from the AC to the subconjunctival space at the equator. The Ahmed implant (AGV) provides a relatively regulated aqueous outflow because of its flow-restricted ‘valve’ design. This reduces the risk of hypotony compared to Baerveldt and Molteno. The Molteno and Ahmed implants are also available in pediatric sizes.

The most commonly used GDDs have a tube linked to a plate that is placed in one quadrant. Baerveldt implants (BGI) necessitate placement of the plate behind the rectus muscles to avoid anterior migration. In cases of associated limbal stem cell deficiency such as aniridia, conjunctival incision should not be performed in the limbal area. The tube may be placed in the AC, sulcus, or pars plana. The optimal length differs according to the location of the tube or specific clinical features; sulcus or pars plana positioning requires longer tube length. This allows the visualization of the tip to be assessed in the case of an occlusion (1). The tube should be short in cases of aniridia to avoid lens trauma or corneal endothelial loss. In contrast, longer tubes may be preferable for uveitis to facilitate occlusion in cases of postoperative hypotony. The long tube is also less prone to occlusion with advancing peripheral anterior synechia.

In addition to the efficiency of GDDs in long-term sustainable IOP control, they have more significant potential for complications than adults, most of which are related to either postoperative hypotony or the tube itself (50). These are more common in patients with aphakia. Hypotony-related complications included shallow AC, hypotony maculopathy, choroidal effusion, suprachoroidal hemorrhage, and phthisis. Potential damage to either the lens or the cornea may lead to iatrogenic cataracts and corneal edema. Reduced scleral rigidity in children, most significantly in buphthalmic eyes, increases the risk of hypotony-related complications. Even with the use of valved implants, peritubular leakage might occur, leading to serious complications such as choroidal effusion and hemorrhage in the suprachoroidal space (51). Intraoperative hypotony is avoided using an AC maintainer and long construction of the limbal tunnel incision. Use of an intraluminal stent or extraluminal ligating vicryl suture with venting *‘Sherwood’* slit anterior to the suture are suggested modifications to avoid postoperative hypotony in non-valved GDDs (52). Leaving the AC with viscoelastic agents at the end of surgery is also useful for the prevention of early postoperative hypotony.

**Figure 5:**
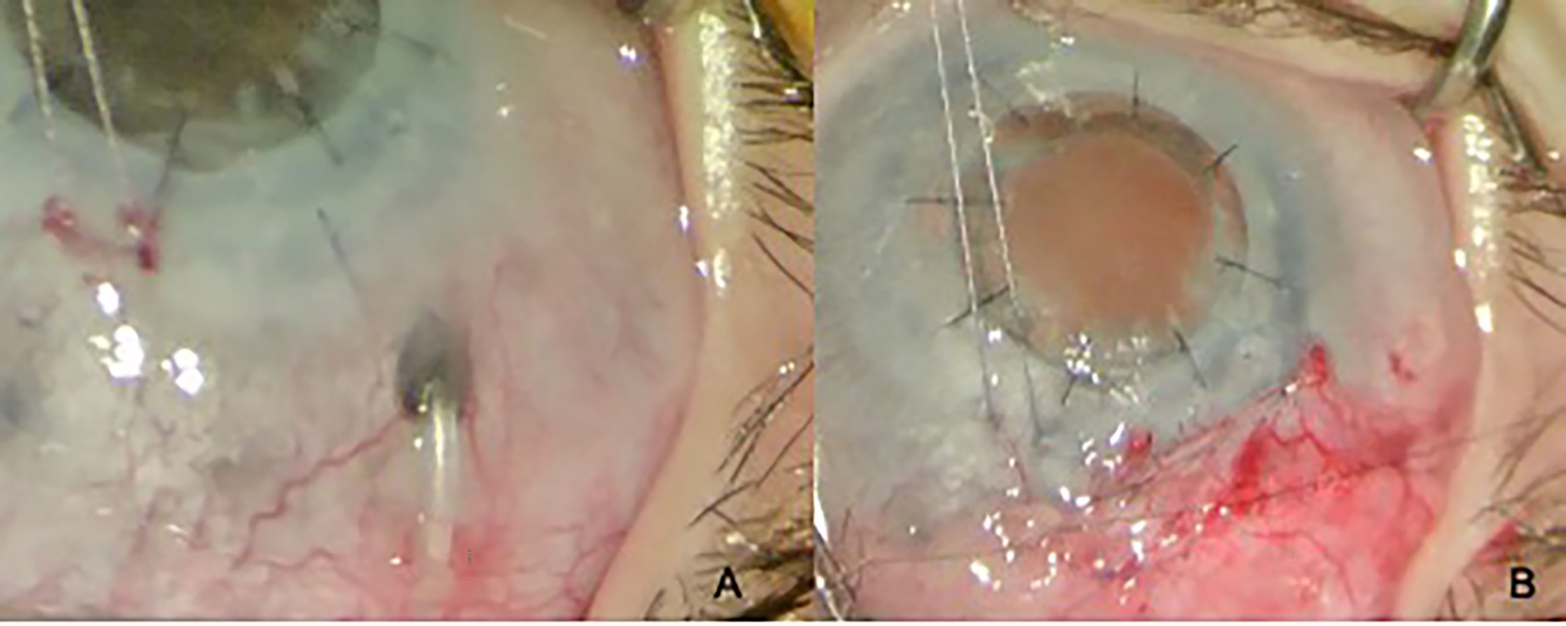
Tube exposure **(A)** and its closure **(B)** in a 2-year-old child with PCG and a history of penetrating keratoplasty.

Other complications associated with tube surgery include extrusion, tip occlusion, migration of the tube, and damage to the iris and/or lens. These consequences often require revision surgery, especially in children under two years of age (**Figures 5A, B**) (53). Together with the normalization of IOP or loose fixation of the plate, it could lead to anterior migration of the tube. Inadequate IOP control, leading to globe enlargement or retraction caused by aggressive healing, may result in posterior migration of the tube. Any contact with the cornea results in corneal decompensation, and iris touch may result in inflammation of the iris and corectopia. The restriction of ocular motility and strabismus should also be considered in children (54, 55).

The reported success rate of GDDs is approximately 80%-90% within 2 years postoperative (56, 57). Unfortunately, this rate falls to approximately 50% during long-term follow-up. The implant type did not seem to influence the success rate (5). Beck et al. compared mitomycin C-augmented trabeculectomy to GDD (AGV or BGI) in pediatric glaucoma patients <2 years old. The success rate was 87 ± 5% for the GDD compared to 36 ± 8% for the trabeculectomy group at 12 months follow-up examination. At 72 months, the success rate was 53 ± 12% in the GDD group compared to 19 ± 7% in the trabeculectomy group (53).

Ahmed ClearPath (New World Medical, Rancho Cucamonga, California, USA) is a novel valveless device introduced in 2019. The device features a more flexible plate and more anterior fixation points, as well as a pre-threaded ripcord (58). The initial results of Ahmed clear path (ACP) glaucoma valveless GDD, implanted in seven eyes of five patients with childhood glaucoma, were revealed by Elhusseiny et al. (59) At the end of first year, complete success was achieved in four eyes (57,14%). Aurolab aqueous drainage implants (AADI) seem to be safer than AGV in terms of early postoperative complications, while both are advantageous over valve-free implants in terms of postoperative hypotony (60).

## 6 Cyclodestruction

Cyclodestruction is preferred in refractory cases with poor surgical prognosis. Cyclocryotherapy has been replaced by laser cyclophotocoagulation because the former is more destructive. Transscleral diode lasers (TSCPC) are preferred to Nd: YAG lasers because they are safer alternatives with better tolerability (61–63). TSCPC should be performed under transillumination in buphtalmic eyes with distorted anatomical landmarks to ensure accurate laser placement. Moreover, areas with pigmentation, hemorrhage, or scleral thinning should be preserved because of the higher risk of scleral perforation (64). Complications associated with continuous wave TSCPC (CW-TSCPC) include conjunctival burns, hypotony, phyhisis, uveitis, cataracts, retinal detachment, scleromalacia, and vision loss. These are reported to be up to 18%, and more common in eyes with successive treatments or in eyes with already low vision (61, 63–66).

An endoscopic diode laser (ECP) provides the treatment location of the ciliary processes *via* the intraocular approach (67). The success rate of TSCPC with continued medical therapy is reported to reach up to 50% with medical therapy, with a repetitive treatment rate of 33-70% (65, 66). Kirwan et al. reported that, among the other secondary glaucomas, aphakic eyes benefit more from a more sustained control; however, ECP seems to achieve similar success and is associated with a lower reproductive treatment rate (65, 67). However, retinal detachment is a potential complication encountered in approximately 9% (3/34) of cases, similar to an endoscopic diode (6%) (65, 68).

The use of micropulse laser cyclophotocoagulation (MP-CPC) in children is relatively new. Recently, Lee et al. compared MP-CPC in adults to that in children. Seven of nine children who were treated with MP-CPC required additional glaucoma surgery within 1 year, which proved that MP-CPC is inadequate for IOP management in children (69). Inadequate success in the pediatric age group (22%) is attributed to the high regenerative ability of the ciliary epithelium in children and the inaccurate localization of the laser beam in buphtalmic eyes with distorted anatomical landmarks (69, 70). In contrast, Abdelrahman et al. reported a 71% success rate in the MP-CPC group, which was sustained at the sixth postoperative month. The authors accounted for these higher success rates to a greater extent of treatment in their study. They also showed a lower success rate in the CW-CPC group (46%) (71). The risk of complications was low in the MP-CPC group. Lee et al. reported mild inflammation detected in the postoperative short-term (69). Aquino et al. conducted an adult study comparing CW-CPC with MP-CPC for refractory glaucoma and reported that the complication rate was lower in the micropulse group (72). The postoperative inflammation rate was higher in the CW-CPC group (30%) than that in the MP-CPC group (4%). Postoperative pain was more frequent in the CW-CPC group than in the MP-CPC group.

To sum up, despite the well-known potential for sight-threatening complications, both CW-TSCPC and ECP seem to be effective in children. ECP appears to be superior to CW-TSCPC in pediatric refractory glaucoma. Unlike ECP combined with cataract surgery, evidence supporting the wider use of CW-TSCPC and MP-TLT in earlier stages of neuropathy is lacking. It seems that the safety profile of MP-TLT is superior to that of CW-CPC (65).

## 7 Recent novel tecniques for childhood glaucoma

### 7.1 Minimal invasive glaucoma surgery

There are various minimally invasive surgical options for the management of pediatric glaucoma:

1. XEN Implant
2. Kahook Dual Blade
3. Gonioscopy-assisted transluminal trabeculotomy (GATT)

MIGS often has the advantage of preserving the conjunctiva, although some of the current MIGS techniques still result in formation of filtering blebs. As childhood glaucoma often necessitates multiple surgeries over the lifetime, conservation of conjunctival tissue during the initial surgery is an advantage for the success of further surgeries. In addition, most of the late postoperative complications of glaucoma surgery are associated with either the implant itself (i.e., corneal edema, motility disturbance, extrusion of the tube, and occlusion of the tube) or bleb (bleb leak, hypotony maculopathy, and endophthalmitis) (73). Thus, blebless and implant-free procedures may be suitable in children. Factors affecting the choice of surgical procedure include clarity of the cornea and the status of the angle (open/unincised, open/incised, or closed).

An electrosurgical trabeculectomy device (Trabectome) and Kahook dual-blade (KDB) are both used for ab-interno trabeculectomy. Similar to goniotomy, both the electrosurgical trabeculectomy device and the KDB device are entered through a clear corneal incision and advanced to the nasal angle. This device engages the trabecular meshwork under direct gonioscopic visualization. The trabectome provides ablation of the trabecular meshwork tissue, whereas the KDB provides simultaneous incision of the anterior and posterior trabecular meshwork. Both devices require direct visualization of the angle; however, they do not allow circumferential treatment of the AC. These devices are assumed to have a lower risk of postoperative fibrotic closure of the trabecular cleft than standard goniotomy (74). They are contraindicated when the visualization of the angle is obscured by a hazy cornea, as in goniotomy. A recent study by Elhilali et al. compared KDB trabeculectomy with conventional goniotomy in 42 eyes of 29 children with PCG at the end of the first year of life (75). The success rate was 57.1% in each group, with no significant complications. According to the results at the end of the first year, KDB is as effective as goniotomy for the management of PCG. Bilateral glaucoma is a risk factor associated with the failure of both procedures. Quiao et al. compared the efficacy and safety of GATT and KDB in juvenile open-angle glaucoma (76). The results favor GATT over KDB in terms of IOP reduction and requirements for additional surgery. This can be attributed to the non-circumferential incision of the angle with the KDB, as opposed to the CT achieved by the GATT.

GATT allows an ab interno incision of the angle circumferentially, leaving the conjunctiva intact without the creation of a scleral flap (77, 78). The main incision and a clear corneal incision were constructed 90° apart. A goniotomy through the main incision was initially performed. Subsequently, a filament (either a blunted suture or an illuminated microcatheter) was inserted *via* the corneal incision and moved through the goniotomy into the Schlemm’s canal using microforceps.

**Figure 6:**
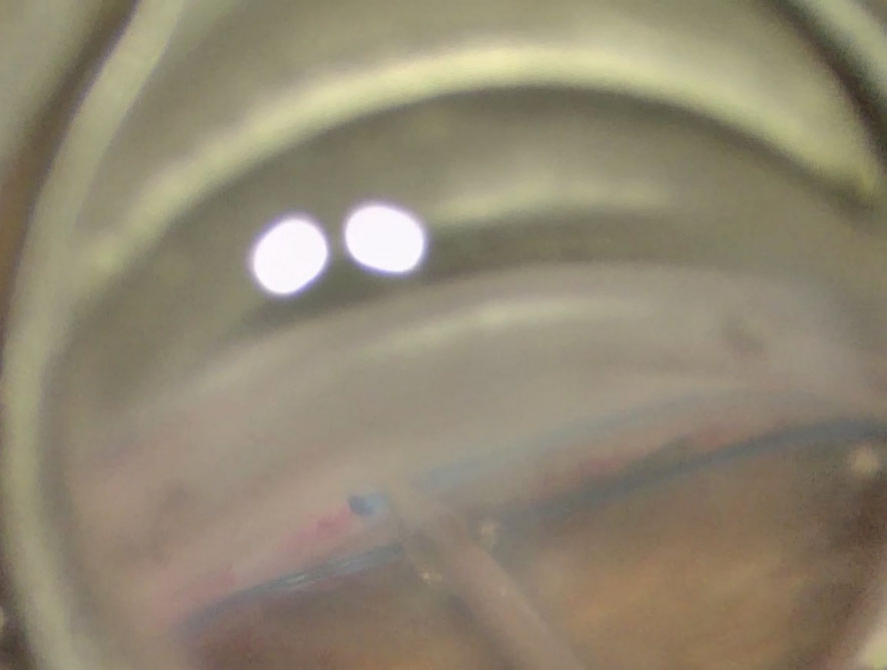
Pulling of the distal part of 5-0 Prolene suture after Schlemm canal is cannulated circumferentially during GATT procedure.

Circumferential cannulation is completed and the distal end of the filament is then drawn, rupturing the inner wall of Schlemm’s canal, which provides a 360° incision of the trabecular meshwork (**Figure 6**). Circumferential intervention of the angle is an advantage of GATT over goniotomy. However, as in goniotomy, GATT is contraindicated unless the cornea is sufficiently clear, providing optimal visualization of the angle. GATT is a relatively new approach for childhood glaucoma; a recent study by Chen et al. investigated the factors associated with complications and failure of GATT surgery in children. In total, 74 eyes were included in the study, with a median follow up time of 28 months, and the success rate was found to be 51.4%. Hyphema was the most common early postoperative complication (48.6%). Non-circumferantial surgery was associated with failure. Additionally, postoperative IOP spikes were found to be correlated with surgical failure. Postoperative use of NSAID or steroid drops were recommended since they were less likely to show postoperative IOP spikes (79).

The XEN gel stent (Allergan, Dublin, Ireland) is an ab interno instrument that creates a shunt between the AC and the subconjunctival space, forming a perilimbal bleb. It may be regarded as a safe alternative for eyes with open angles following failed angle surgery. A fixed and tight channel size decreases the risk of hypotony. However, long-term results compared with trabeculectomy in children have not yet been reported. Smith et al. reported a series of PCG cases that were treated with XEN gel stent implants. No device-related complications were noted (80). The IOP was controlled without the use of topical medications for 6–24 months of follow-up.

The Preser-Flo ^®^ MicroShunt (PM) is a stent made of polystyrene-block-isobutylene-block-styrene (SIBS) and implanted ab externo through a constricted conjunctival flap and a small scleral tunnel (81). Open-angle glaucoma, which cannot be controlled by medications, is the main indication. Complications are reported to be rare; transient hypotony, shallow AC, choroidal detachment, and hyphema (81–85). Preserflo microshunt (PM) implantation requires conjunctival incision and the creation of a scleral pocket. The most important advantage of this device is the durability of the device, as children with glaucoma are expected to live with the disease for a lifetime. Widder et al. recently reported a case of intraocular degradation of a XEN gel stent 3 years after its implantation (86). Unlike the collagen-derived gelatin XS material cross-linked with glutaraldehyde, the PM material “SIBS” is a recently developed thermoplastic biomaterial that resists biodegradation, which is a theoretical advantage over the XEN gel stent (87). In contrast, the first generation of micro-shunts showed a high rate of erosion, leading to modified surgery with the creation of a scleral pocket (85, 88).

Preser-Flo ^®^ may be advantageous in terms of its SIBS material in children. On the other hand, all devices mentioned here are off-label for children. A clinical study of PM in children was reported recently in the US. In total, 12 eyes of 12 children were included and the success rate at the end of the first year was 75%, without any surgical complications recorded (89).

## 8 Discussion

Childhood glaucoma is a challenge, necessitating perfect timing and selection of the most appropriate surgical approaches and life-time follow-ups. Therefore, an intensive search for improved and more optimal alternative methods is ongoing. The vast majority of studies in the current literature have focused on PCG, since it is a major cause of blindness in the pediatric age group, presenting by 1 year of age in approximately 70% of patients and accounting for up to 18% of the causes of blindness in children (88, 90, 91). Since medical treatment for glaucoma in pediatric patients is less effective than that in adults and poorly tolerated, the definitive treatment for congenital glaucoma (trabeculodysgenesis) is surgical (4). The traditional first-line approach in the literature is angle surgery, either goniotomy or trabeculotomy (traditional or circumferential).

Owing to the high success rates of both goniotomy and trabeculotomy, angle surgery remains as an initial choice in mild cases. Among these, CT has been shown to be a safer and more effective procedure than TT for the management of PCG in numerous studies. Another important advantage seems to be the sustainability of CT over TT. Aktas et al. stated that the IOP-lowering effect of CT could be maintained longer than that of TT (32). Consistently, El Sayed et al. reported the need for subsequent glaucoma procedures to be 10% in the microcatheter group and 41% in the conventional group; therefore, the conventional group had significantly more cases that needed re-operation than the circumferential group (92). Primary CTT for PCG is more often preferred in certain ethnic populations (such as India and the Middle East) since they present earlier and have a more severe phenotype (India and Middle East). In these cases, combined surgery offers better IOP control than trabeculotomy or trabeculectomy alone (10, 93–96). However, a recent study from India suggested that CT with an illuminated microcatheter provided success rates comparable to that of primary CTT with MMC. Unfortunately, the high cost of microcatheters prohibits their widespread use in developing countries (96). For the time being, based on these results, transition to CT or primary CTT with MMC as the first-line treatment for PCG and some secondary glaucomas might be an attractive alternative. However, congenital anomalies or scarring from previous surgeries, which prevent full canalization, may inherently decrease the success rates of angle surgery in other glaucoma (97).

Refractory glaucoma involves cases that fail a primary surgical procedure, those that have an inadequate response to the angle surgery, and those with complex glaucoma (ie., uveitic glaucoma, glaucoma following cataract surgery, and anterior segment anomalies). Trabeculectomy with or without MMC is preferred in these circumstances. GDDs have a good success rate in the treatment of aphakic eyes. The Ahmed implant (63%) and Baerveldt implant (41%) are the most preferred implants (1). In addition to the early postoperative tube-related complications, these devices may later fail because of globe enlargement, leading to tube retraction into the corneal stroma or outside the AC. This results in a decrease in the IOP-lowering effect of surgery over time, necessitating a better drainage device for childhood glaucoma. Transscleral cyclophotocoagulation may be an additional intervention unless IOP is adequately controlled by shunt implantation (1).

Diode laser cyclophotocoagulation is recommended in cases with a high-risk of failure or where incisional filtering surgery or GDD has already failed. The status of conjunctival and subconjunctival tissues should be considered. The inability to ensure strict follow-up should also be considered (American Academy of Ophthalmology recommendations [2010]). Diode laser cyclophotocoagulation is preferred in the eyes with silicone oil tamponade following surgery for retinal detachment (39, 98). Uncontrolled IOP in eyes with poor visual acuity is another common indication for laser cyclophotocoagulation. Other surgical procedures or sudden decompression of the eyeball always have a greater risk of visual loss than cyclophotocoagulation. Cyclophotocoagulation can be used as a palliative measure for blind or visually impaired eyes with advanced stages of glaucomatous neuropathy. Patients who would not tolerate incisional surgery due to a poor general condition, low adhesion to the follow-ups, and acute neovascular glaucoma (NVG) are other indications for cyclophotocoagulation. For refractory glaucoma, augmentation of the IOP-lowering effect with diode laser cyclodestruction following tube implantation has been proposed by some authors, with a low level of evidence (99).

In summary, diode laser cyclophotocoagulation has inadequate long-term success rates and often requires successive treatment and continuation of glaucoma medications with potential vision-threatening complications. ECP has a similar success rate and is superior in terms of predictability and sustainability, thus an invasive measure necessitating an intraocular approach. Extended (four quadrants) application of MP-TLT would be a safer and more effective alternative; however, confirmation with prospective comparative studies is still needed (100).

MIGS has been changing the traditional approaches in adults and has proven to be efficient in mild to moderate glaucoma, but the role of MIGS in childhood glaucoma has not yet been fully explored. These advances and recent devices of MIGS surgery have begun to be integrated into the management of childhood glaucoma, starting with PCG. In the light of recent studies, GATT and KDB provide a safer, efficient, and sustainable alternative among MIGS procedures, either for cases failing angle surgery or as the primary approach for PCG. Future studies are needed to evaluate the use of the Preser-Flo ^®^ in children.

In conclusion, owing to the higher success rates and longer sustainability and safety, most pediatric glaucoma surgeons currently prefer CT as their initial treatment approach. The introduction of an illuminated catheter makes this procedure more predictable and safer. Circumferential ab externo trabeculectomy is another option for patients with opaque corneas and can be performed inexpensively with a Prolene suture. Circumferential ab interno trabeculotomy, on the other hand, avoids violation of the conjunctiva and preserves it for later surgeries. However, the view of the angle is often suboptimal and the technique has a steep learning curve, which makes it more challenging. In addition, the procedure is associated with expensive devices and consumables.

Although considerable progress has been made in the management of congenital glaucoma, it remains a potential cause of blindness, requiring lifelong follow-up. Therefore, further innovative, well-designed randomized controlled trials are required to improve the existing medical and surgical options for the treatment of childhood glaucoma.

## Author contributions

Authors have contributed equally in the process of planning, research, construction and revision of this review. The figures all belong to the surgical cases of ZA.
